# Oral health-related interdisciplinary practices among healthcare professionals in Saudi Arabia: Does integrated care exist?

**DOI:** 10.1186/s12903-022-02113-5

**Published:** 2022-03-17

**Authors:** Balgis Gaffar, Faraz Ahmed Farooqi, Muhammad Ashraf Nazir, Eman Bakhurji, Khalifa S. Al-Khalifa, Muhanad Alhareky, Jorma I. Virtanen

**Affiliations:** 1 Department of Preventive Dental Sciences, College of Dentistry, Imam Abdulrahman Bin Faisal University, Dammam Costal Street, B.O Box 1982, Dammam, Costal Code 31441 Saudi Arabia; 2 Department of Dental Education, College of Dentistry, Imam Abdulrahman Bin Faisal University, Dammam, Saudi Arabia; 3 Department of Clinical Dentistry, University of Bergen, Bergen, Norway; 4 Faculty of Medicine, University of Turku, Turku, Finland

**Keywords:** Knowledge, Attitudes, Interprofessional, Interdisciplinary practices, Integrated care, Oral health

## Abstract

**Background:**

There is a bidirectional relation between oral and general health, therefore collaboration between healthcare providers is needed. This study investigated current interdisciplinary practices (IDP) and the associated factors among healthcare professionals in Saudi Arabia.

**Methods:**

A cross-sectional study was conducted in the Eastern Saudi Arabia recruiting four groups of health professionals (nurses, physicians, pediatricians and Ear-Nose and Throat (ENT) specialists). A validated, self-administered questionnaire was distributed online and shared through social media platforms. The questionnaire explored predisposing factors (demographics) and facilitating factors (knowledge, attitudes, attendance of oral health training and source of knowledge) associated with IDP.

**Results:**

A total of 1398 health professionals were recruited. Participants showed fair oral health knowledge (7.1 ± 2.1) and attitudes (22.2 ± 3). Three-fourths (74.6%) reported always providing oral health education (OHE) to their patients, more than half (59.6%) reported always conducting an oral health screening (OHS), two-thirds (66.7%) reported responding to patients’ questions about oral health or conditions and 58.7% reported referring patients to dentists. Pediatricians and physicians had greater odds of IDP compared to other health professionals. Source of oral health knowledge (Ministry of Health (MOH) and formal education) was significantly associated with increased odds of IDP. Participants with good oral health knowledge had greater odds of responding to patients’ oral health question as well as have more referral practices.

**Conclusion:**

The results reveal a discrepancy between participants' IDP, knowledge, and attitudes. Incorporating dental component to medical curricula, continuous education and training programs targeting health professionals through Ministry of Health should be considered.

## Background

The first holistic understanding of health came from the World Health Organization (WHO) which defined health as “a state of complete physical, mental, social, and spiritual well-being and not only the absence of disease or infirmity” [1]. Likewise, oral health encompasses physical, psychological, emotional, and social aspects that are integral to the overall general health [2]. Oral diseases are prevalent and could result in a significant burden on children and adults worldwide. Although preventable, they are still a burden in many countries and their related factors include cost of dental care, barriers accessing care, lack of screening and monitoring as well as patient factors [2, 3]. On the other hand, there is a mutual bidirectional relationship between oral and general health, as many systemic diseases have oral symptoms whereas poor oral health can aggravate many medical conditions [4].

Integrated care (IC) is defined as “bringing together inputs, delivery, management and organization of services related to diagnosis, treatment, care, rehabilitation and health promotion” [5]. Besides lowering health care costs, IC has been promoted by the WHO to improve health outcomes [6]. Integrating health care supports the collaboration of different health professionals and sectors as such improving treatment outcomes and the utilization of healthcare services for all individuals regardless of their socioeconomic status [7]. Integrating public and private partnerships, interprofessional education, and collaborative practice are examples of integration at various levels [8, 9]. Some studies have evaluated integrated health practices among different health and dental care providers [8, 9]. However, macrolevel (supportive policies, system domains of integration, budget allocation, interdisciplinary education and training), meso-level (organizational setup, working environment, time constrains, shortage of resources and manpower) and microlevel (knowledge, attitudes, perspectives, beliefs, and values) were factors that hindered such integration [8–11]. For instance, Smith et al. [11] developed a model explaining the barriers and enablers to extended scope of practice among nurses. In the same context macrolevel factors such as perceived legal, regulatory, and financial support affect the setup within the different organizations, the recruitment of manpower and the financial compensations for extra time and tasks (meso-level factors). These higher level and organizational factors influence healthcare providers attitudes and facilitate interdisciplinary practices [11].

In Saudi Arabia a couple of studies investigated oral health knowledge among health professionals. Alshathri et al. reported that only 7% of family physicians in Saudi Arabia received oral health training and that referral practices to dental clinics were also low [12]. Similarly, Almazrooa et al. observed a substantial lack of knowledge among family physicians regarding oral health and reported that only 42% of Saudi Arabian medical practitioners would request dental consultations for their patients prior to bisphosphonate medication, indicating a lack of knowledge of the maxillofacial consequences of bisphosphonate therapy and the need for special dentalcare [13]. In the same context, Zakirulla et al. [14] found that oral health knowledge of nurses working in pediatric intensive care units in southern Saudi Arabia was proportional to their level of education; and although the majority of the nurses agreed that getting proper oral care is important for the overall health and wellness, yet they claimed lack of education, lack of time and workload as potential barriers [14].

Saudi Health Information Survey (SHIS) found that only 11.5 percent of Saudi Arabia's population aged 15 and above visited the dentist regularly, and half the visits were driven by dental complains [15]. The main barriers for underutilization of dental care among children are oral health illiteracy, financial, dentist-related (unsuitable appointment times and long waiting time) and transportation [16]. Despite the fact that the Ministry of Health (MOH) provides most dental services for free the burden of oral diseases in Saudi Arabia is still high [17]. Dental caries prevalence was found to be 80% in the primary dentition and 70% in the permanent dentition [18]. While the prevalence of periodontal diseases was estimated to be 90% among residents aged 25 and over [19].

Healthcare systems demand well-trained specialists that cooperate interprofessionally to deliver comprehensive and ongoing treatment to effectively improve the health and quality of life of each patient. It has been proposed that integrated oral care utilizing and expanding the role of healthcare providers can lead to improvement of oral health, reduction of burden of oral diseases, and addressing oral health disparities [20, 21]. Therefore, this study aimed to investigate current interdisciplinary practices and the associated factors among healthcare professionals in Eastern province of Saudi Arabia.

## Methods

### Study design and setting

This cross-sectional survey was conducted during the period from October to December 2020 in the Eastern Province of Saudi Arabia.

### Study participants

The Eastern Province of Saudi Arabia has a total population of almost 4 million according to the 2016 MOH report, an approximate number of 4025 physicians and 8182 nursing staff [22]. The study targeted nurses, physicians, ear-nose and throat (ENT) specialists and pediatricians in the Eastern Province of Saudi Arabia. The study team searched contact information of the targeted groups within the Eastern Province public, private and teaching institutions’ websites. The data collection was carried during the first wave of the COVID-19, and the country was in partial lockdown and access to hospitals and healthcare facilities was difficult as well as locating possible participants. We used exponential non-discriminative snowball sampling. We reached out for initial participants through emails and then requested their help in sharing the survey link with doctors and nurses within their workplace or personal connections, who in turn shared it through their network. The survey link was open from October to December 2020 and a purposive sample of the four groups of health professionals was recruited. Participants who were working in the Eastern Province during the study duration and who agreed to participate in the study were included. There were no exclusion criteria.

### The questionnaire

A structured, self-administered questionnaire was distributed online using google forms and shared via different social media platforms (WhatsApp and Twitter). The questionnaire was adopted from previous studies [23–26] and modified by the authors for the purpose of this study. The questionnaire was delivered in English and Arabic to ensure maximum clarity and easiness. The English version of the questionnaire was translated into Arabic and then back translated to ensure accuracy of the translation. The questionnaire included 40 closed-ended questions and was divided into four sections (demographics, oral health knowledge, attitudes towards oral health, and IDP related to oral health). The questionnaire was pilot tested on 20 health professionals who were not included in the study. The Cronbach's Alpha for the whole questionnaire was 0.816.

#### Outcome variables

Participants’ IDPs were assessed through four questions. The participants were asked if: (1) they provide oral health education (OHE) to their patients with answer options of always, sometimes, or never. (2) They perform oral health screening (OHS) with answer options always, sometimes, or never. (3) They respond to patients’ questions about an oral health condition, with two answer options yes or no (4) they have previously referred a patient to dentist; with two answer options yes or no.

#### Predisposing factors

These were the participants’ demographic data and background. It included (1) Age (20 -29 years old, 30–39 years, 40–49 years old, or 50 years old and above). (2) Sex of participants (male and female). (3) Nationality (Saudi or Non-Saudi). (4) Specialty (nurses, physician, pediatrician, or ENT specialist). (5) Affiliation (public hospital, MOH, teaching institute, private hospital, or both private and public). (6) Years of working experience (< 3 years, 3–6 years, > 6 but < 10 years, or > 10 years).

#### Facilitating factors

These factors included (1) Participants’ oral health knowledge assessed through thirteen questions. Knowledge about the common dental diseases (dental caries, gingivitis and periodontitis), their risk factors, if these were preventable, and their consequences. (2) Participants’ attitudes towards oral health and oral healthcare provision assessed through fourteen statements. Participants responded to each statement on a 5-point Likert scale: strongly agree, agree, neutral, disagree or strongly disagree. (3) Attendance of continuous educational sessions or training related to oral health and care, answered as yes or no. (4) Source of participants’ oral health knowledge, participants could choose one or more options (formal/college education, social media, scholarly publications, MOH or choose I do not have any dental or oral health knowledge).

### Statistical analysis

#### Scoring of knowledge section

For questions with a single correct answer, a score one was given for each correct answer and zero for wrong or “I do not know” answers. For questions with multiple correct answers, the whole set of correct answers was scored as “one” while choosing one or two correct answers was given “zero”. The individual’s total knowledge score was the sum of points for all correct answers yielding a maximum score of thirteen. Participants were labeled as having a good knowledge if they have an overall score of 70% and above, answering half of the questions correctly would be considered average, while scoring below that would be considered poor knowledge [27].

#### Scoring of attitude section

For the items included in the attitude section agree and totally agree responses were combined and disagree and totally disagree responses were also combined. We measured the participants' attitude by calculating the mean score of attitude statements, and the participants were considered positive or negative based on whether their score exceeded the mean score or fell below it [27].

Descriptive statistics (frequencies, percentages, mean, median, and standard deviations [± SD]) were calculated. Data was checked for normality using Shaprio Wilk test. P-values less than 0.05 suggested a significant deviation of data from normality. For comparisons of knowledge and attitude scores among the healthcare specialties a Kruskal Wallis Test (non-parametric ANOVA) was used and for inter group comparisons a Bonferroni Correction method was applied. A logistic regression analysis with backward elimination was used to identify factors associated with participants’ oral health related IDPs. Odds ratio (OR) with 95% confidence interval was computed. A *P*-value of less than 0.05 was considered statistically significant. The data was analyzed using the SPSS version 23 software.

## Results

A total of 1,398 health professionals responded to the survey, of whom 537 (39%) were between 20 and 29 years, 873 (63%) were males, 1062 (76%) were Saudis, and 525 (38%) were working in health centers. Table 1 shows that most of the participants were nurses 525 (38%) and 478 (34%) had less than 3 years of working experience. From the participants about half (48.5%) depended on their formal (university/college) education as a source of oral health knowledge, and 862 (61.7%) had never attended any oral health lecture or training.

**Table 1:** Background description of the study sample (n = 1398)

Variables	Frequencies (%)
**Age**	
Twenties	537 (39)
Thirties	510 (36)
Forties	226 (16)
Fifties and above	125 (9)
**Gender**	
Male	873 (63)
Female	523 (37)
**Nationality**	
Saudi	1062 (76.0)
Non-Saudi	335 (24.0)
**Health professionals**	
Nurses	525 (38)
Physicians	303 (22)
Pediatricians	329 (23)
ENT specialists	161 (12)
Others	80 (6)
**Affiliations**	
Public hospitals	275 (20)
Health centers	525 (38)
Teaching institutes	192 (14)
Private hospitals	298 (21)
Both private and public	105 (8)
**Years of experience**	
< 3 years	478 (34)
3–6 years	322 (23)
7–9 years	264 (20)
≥ 10 years	333 (24)
**Source of oral health knowledge**	
Formal education	679 (48.6)
Social media	522 (37.3)
Scholarly publications	411 (29.4)
Ministry of Health	198 (14.2)
No previous knowledge	350 (25)
**Have you attended an oral health lecture or training?**	
Yes	511 (37.2)
No	862 (61.7)

**Fig. 1:**
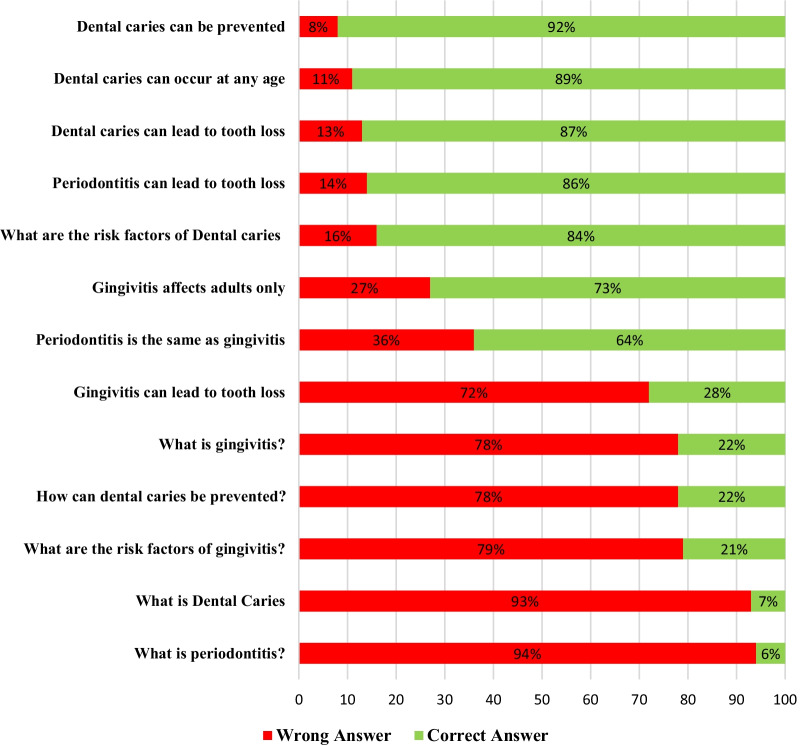
The healthcare professionals’ knowledge of basic oral health related questions/topics

Figure 1 presents the participants’ responses to oral health knowledge questions. Most of the health professionals were unaware about the clinical description of dental caries and periodontal disease; only (7%) and (6%) provided the correct answers respectively. The participants had good knowledge that dental caries (87%), and periodontal disease (86%) can lead to tooth loss.

**Fig. 2:**
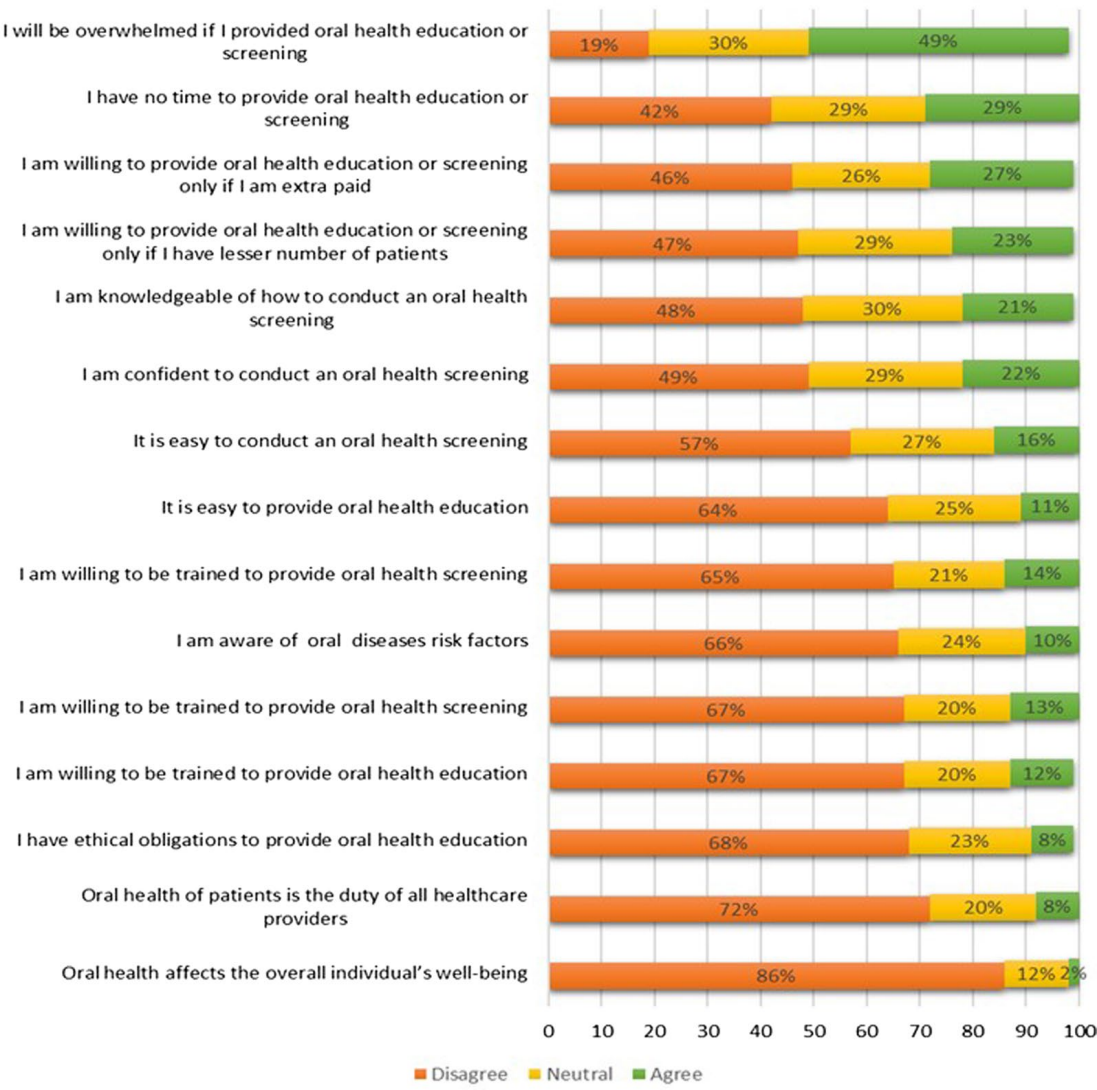
Responses of health professionals with regards to attitude statements

Figure 2 shows the health care professionals responses to the attitude statements. Most of the respondents (86%) disagreed that oral health affects the overall wellbeing of individuals. Almost half of them (49%) agreed that providing OHE would be a burden. Two-thirds (67%) of the health professionals were willing to receive training to provide OHE and screening.

Table 2 shows the knowledge and attitude scores among healthcare professionals. The mean score of oral health knowledge among health professionals was (7.1 ± 2.1). The knowledge scores among the healthcare professionals varied significantly (*p* = 0.0001). Physicians had the highest overall oral health knowledge score (7.46 ± 2.2) while the lowest knowledge score was reported by the nurses (6.8 ± 2). The overall mean score of participants’ attitudes towards oral health among health professionals was (22.2 ± 3). The healthcare professionals’ attitudes towards oral health varied significantly (*p* = 0.0001) among the specialties; the most positive attitudes were observed among ENT specialists (23.3 ± 2.8) and lowest was observed among the nurses and it differ significantly.

**Table 2:** Comparison of knowledge and attitude overall score among the different specialties

Affiliations	Knowledge score	*p*-value	Attitude	*p*-value
Nurse	6.8 + 2.04^a^	0.001*	21.46 + 2.88 ^a^	0.001*
Physician	7.46 + 2.2^a^		22.52 + 3.22 ^a^	
Pediatrician	7.22 + 2.16 ^a^		22.47 + 3.08 ^ab^	
ENT	7.24 + 2.1		23.25 + 2.79^ab^	
other	7.35 + 2.21		21.88 + 2.56	

^a,b^Same alphabet showing significant difference between the groups

*Significant at 0.05

**Fig. 3:**
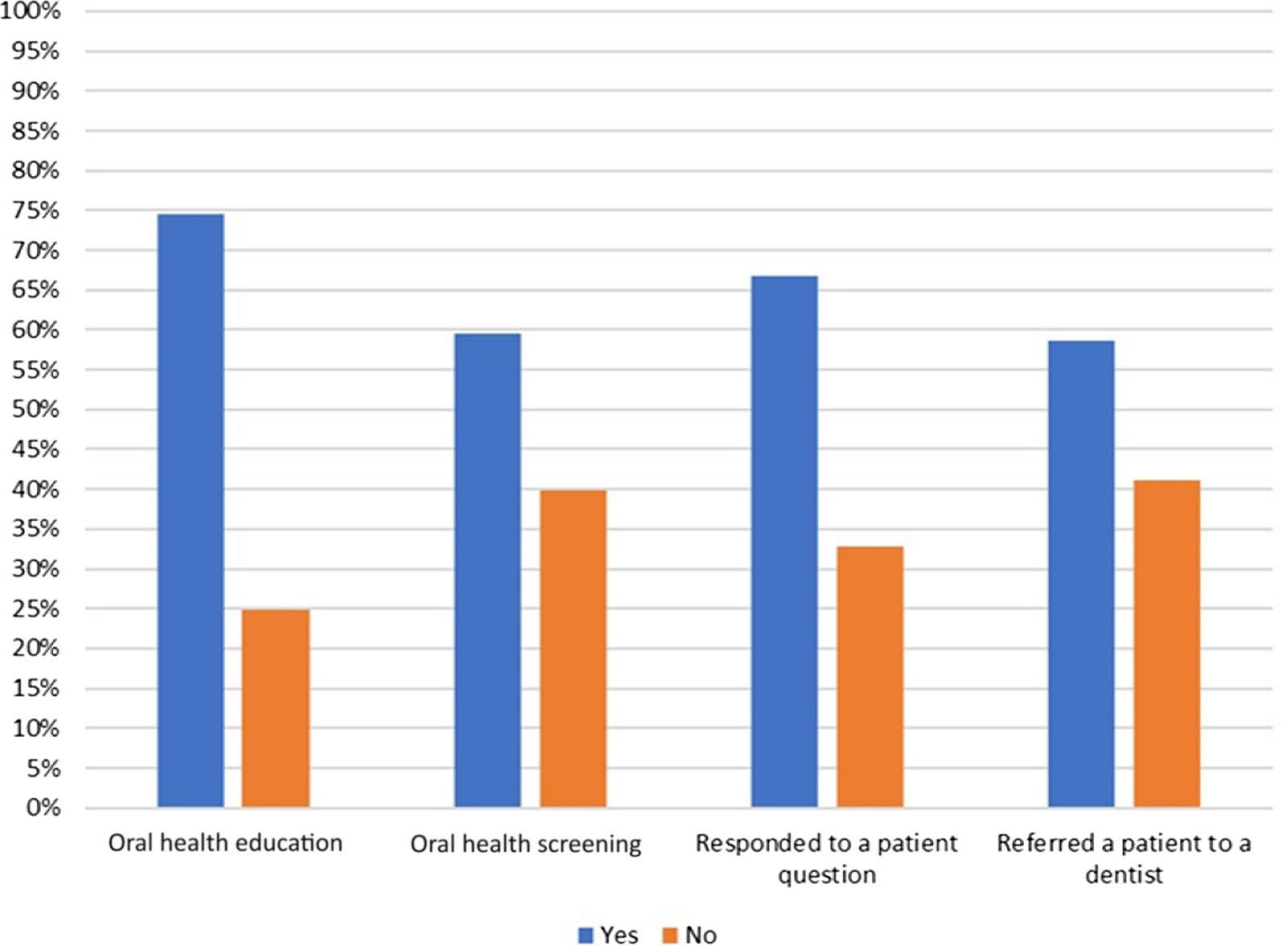
Oral health-related interdisciplinary practices as reported by the study participants

The frequency of interdisciplinary practices as reported by healthcare professionals is presented in Fig. 3. Three-fourths (74.6%) of participants reported always providing OHE to their patients. Similarly, more than half (59.6%) reported always conducting an OHS. Two-thirds of the participants (66.7%) reported responding to patients’ questions about oral health or conditions related to it and 58.7% reported referring patients to dentists previously.

**Fig. 4:**
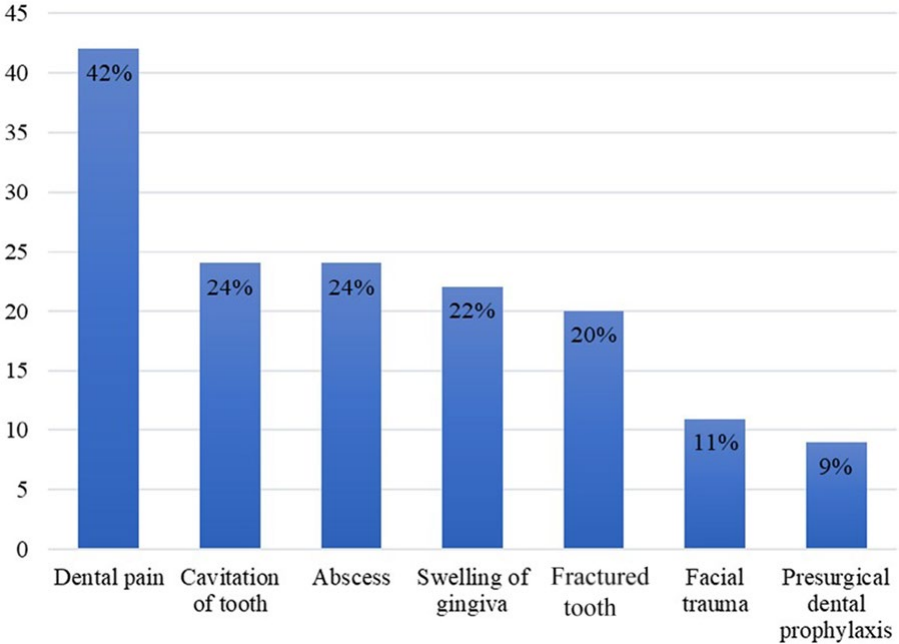
Distribution of health professionals’ responses about the reasons for referral to the dentist

Figure 4 shows the reasons for dental referrals as reported by the participants with dental pain being the main cause of referrals (41.5%).

Table 3 shows factors associated with interdisciplinary practices. Being a pediatrician (OR = 2.6), having oral health knowledge from formal education (OR = 1.8) and having MOH affiliation (OR = 3.6) were significantly associated with greater odds of providing OHE to patients. Physicians (OR = 0.5), ENT specialists (OR = 0.4) and those attended oral health education training (OR = 0.17) were statistically significantly less likely to conduct OHS. Those with good knowledge (OR = 1.1), having more than 10 years of working experience (OR = 1.6), source of knowledge from formal health educational training had statistically significantly greater odds of oral health related interdisciplinary practices (respond to patient’s oral health questions and provide referrals to dentists).

**Table 3:** Factors associated with interdisciplinary practices among the study participants using multiple logistic regression model. (n = 1398)

Independent variables	Oral health- related Interdisciplinary practices					
	OHE		OHS		Respond to PT	Referral
	OR (95% CI)					
**Gender**						
Female	1		1		1	1
Male	0.646* (0.47–0.88)		0.708 (0.54–0.93)		0.883 (0.67–1.16)	0.864 (0.67–1.12)
**Nationality** Saudi vs non-Saudi	0.666* (0.44–1)		1.155 (0.82–1.62)		0.917 (0.65–1.3)	0.683* (0.49–0.95)
**Specialty**						
Nurse	1	1		1		1
Physicians	0.317* (0.22–0.46)		0.454* (0.32–0.64)		0.91 (0.64–1.3)	1.947* (1.38–2.74)
Pediatrician	2.631 * (1.66–4.17)		1.147 (0.81–1.62)		1.081 (0.76–1.53)	2.395* (1.71–3.36)
ENT specialist	0.471* (0.29–0.77)		0.441* (0.28–0.69)		0.587* (0.38–0.92)	0.899 (0.59–1.37)
Others	0.361 * (0.19–0.68)		0.668 (0.37–1.21)		0.665 (0.37–1.2)	1.074 (0.63–1.83)
**Affiliation**						
Public Hospitals	1		1		1	1
Ministry of health	0.726 (0.39–1.34)		1.694 (0.98–2.94)		0.669 (0.39–1.15)	0.971 (0.59–1.61)
Teaching institute	0.844 (0.47–1.5)		1.231 (0.74–2.05)		0.967 (0.58–1.61)	1.533 (0.95–2.46)
Private hospital	0.988 (0.51–1.9)		0.857 (0.48–1.52)		1.132 (0.63–2.04)	0.85 (0.5–1.45)
Both private and public	0.511* (0.28–0.94)		0.884 (0.51–1.52)		0.49* (0.29–0.84)	0.55* (0.33–0.91)
**Years of experience**						
Less than 3 Years	1		1		1	1
3–6 years	0.766 (0.52–1.14)		1.284 (0.91–1.81)		1.038 (0.74–1.46)	1.654* (1.19–2.29)
More than 6 less than 10 years	0.724 (0.47–1.11)		1.062 (0.73–1.55)		0.907 (0.62–1.32)	1.688* (1.18–2.42)
More than 10 years	0.83 (0.55–1.26)		0.986 (0.69–1.41)		1.55* (1.06–2.26)	2.581* (1.81–3.67)
Knowledge score	1.018 (0.95–1.09)		0.917* (0.86–0.98)		1.095* (1.03–1.17)	1.118* (1.05–1.19)
Attitude Score	0.935* (0.89–0.98)		0.916* (0.88–0.96)		0.905* (0.87–0.95)	0.955* (0.92–0.99)
**Attended an oral health education**						
No	1		1		1	1
Yes	0.182* (0.13–0.26)		0.172* (0.13–0.23)		0.249* (0.18–0.34)	0.385* (0.29–0.5)
**Source of knowledge**						
Formal education						
No	1		1		1	1
Yes	1.838* (1.36–2.48)		1.563* (1.21–2.02)		1.652* (1.27–2.15)	1.224* (0.96–1.57)
**Media**						
No	1		1		1	1
Yes	0.702* (0.52–0.95)		0.955 (0.73–1.25)		0.873 (0.67–1.15)	0.85* (0.66–1.1)
**Publications**						
No	1		1		1	1
Yes	1.012 (0.73–1.41)		0.98 (0.73–1.31)		1.213 (0.9–1.63)	0.988* (0.75–1.3)
**Ministry of Health**						
No	1		1		1	1
Yes	3.567* (2.1–6.06)		1.456* (1.01–2.11)		1.714* (1.15–2.56)	1.864* (1.29–2.69)
No dental or oral health knowledge	0.875 (0.62–1.23)		1.478* (1.089–2.006)		1.375 (1–1.89)	0.866* (0.65–1.16)

*Significant at 0.05 level

*OHE* oral health education, *OHS* oral health screening, *OH* oral health, *PT* patient, *CI* confidence interval

## Discussion

This study highlights the gaps in oral health-related interdisciplinary and pointed out the influencing factors among four groups of health professionals in Saudi Arabia. Our results showed that from the predisposing factors, the type of specialty (Pediatricians or Physicians) and years of experience (more than 3 years) were associated with higher odds of referral practices. From the facilitating factors, source of oral health knowledge (MOH and formal education) was significantly associated with greater odds of all interdisciplinary practices. While participants with good oral health knowledge were more likely to respond to patients’ oral health questions as well as have more referral practices. However, attending OHE training and participants’ attitudes were less likely to influence oral health-related interdisciplinary practices.

Participants in the current study demonstrated fair oral health knowledge, which was significantly different among the health professionals with physicians attaining the highest knowledge scores while the nurses had the lowest knowledge scores. Poor oral health knowledge among nurses and better knowledge among physicians have also been reported in other studies [11–14, 23, 25, 26, 28]. The low level of oral health knowledge observed among health professional has been found to be related to limited integration of oral health content in under-graduate training programs [28, 29], as well as the lack of protocols and regulations to meet current standards of integrated care [28, 30]. Policy makers in higher education may consider oral health integration into medical and nursing curricula, and that some sessions be delivered along with dental students.

Almost all participants in the current study were unaware about the clinical presentations of dental caries and periodontitis which is a remarkable finding. However, low competency level for identifying dental caries and oral pathology has also been reported earlier [23, 31]. In our study we had strict scoring criteria for the knowledge section. For example, the question about the clinical presentations of dental caries included white spot lesions, staining as well as cavitation. Only those who were able to choose the whole set of correct answers were considered knowledgeable about the clinical manifestations. The connection between oral and general health is well and undeniably established [4]; physicians, dentists and nurses can be encountered by medical conditions that aggravate oral problems and vice versa [31]. For instance, diabetes and periodontal disease are mutually dependent since both negatively affect each other and share a common pathophysiological pathway [32]. In a country (KSA) where the prevalence of uncontrolled type II diabetes is among the highest in the world (77.7%) [33], oral health knowledge and dentalcare is, therefore, essential. In the same way early childhood caries (ECC) is a prevalent oral health problem with lifelong consequences that affect the child’s health and wellbeing [34], with financial burden on parents as it often/frequently necessitates the aid of general anesthesia [35]. Suboptimal knowledge about early signs of dental caries amongst health care providers may cause delayed referrals and place children at high risk of worsening health and chronic illness [36, 37]. Therefore, it is essential to educate healthcare providers about the early signs and clinical presentations of oral diseases.

The observed attitudes towards oral health and oral health-related interdisciplinary practices were also average in the current study with ENT specialists showing the most positive attitudes while the most unfavorable attitudes were among the nurses which contradicts the findings reported from Riyadh, Saudi Arabia [29] and from the USA [31]. The importance of nurses in maintaining the dental health and well-being of hospitalized patients cannot be overstated [23, 24]; with this in mind, nurses must be willing to provide adequate oral care in their setting. Some of the observed participants’ negative attitudes were concerns about time allocation as well as financial compensations. ENT specialists, for instance, have a scheduled number of patients per day and they have the freedom to allocate the time for each patient. This is not the case for nurses who have no control on the flow of patients or the time, thus it is understandable that another task (such as oral healthcare) would be reluctantly accepted. The first step in changing attitudes is by creating a positive social change which could be achieved by improving the undergraduate curricula, providing training and workshops, presence of a policy for oral care in workplace and equipping the health care setup. Perceived organizational support, or employee impressions of how much their employer values their contributions and cares about their well-being, can influence work attitudes and readiness [38, 39].

Provision of OHE was the most common reported interdisciplinary practice followed by responding to a patient’s question about oral health condition or problem and the least practiced were conducting OHS and referring a patient to a dentist. Referral of patients requires the knowledge and understanding of referral loops within one’s institution which in turn requires extra administrative duties. In the same context, conducting OHS requires confidence which is usually based on sound knowledge (which was not the case in the current study). Studies conducted worldwide among physicians and other healthcare providers have found that education, risk assessment, and referral practices are relatively uncommon [29, 31, 32, 38]. The current study found that the rates at which providers reported engaging in transdisciplinary activities varied by provider type. Pediatricians were twice as likely as family physicians to provide OHE and refer patients to dentists, which is consistent with a previous study that found in terms of general dental knowledge and preventive oral health counseling, pediatricians were better informed than family physicians [40]. Early dental visit is one of the foundations for promoting the oral health of a child and in the prevention of ECC, according to the American Academy of Pediatric Dentistry [41]. Physicians and nurses can be patients' first exposure to health-care system [42]. Children are exposed more to pediatricians and family physicians at earlier age than dentists [38, 40], and they can perform OHS seven times more frequently than dentists [31]. It is important for pediatricians and general practitioners to understand their role in children's oral health, prompt management and referral to dental specialty [23, 43]. If equipped with the essential knowledge, pediatricians can play a major role in educating parents about children’s dental health, caries preventive measures as such help in establishing early dental homes.

It was observed that those with more than 10 years of experience had greater odds of referral practices, similarly those with good knowledge scores were more likely to respond to patients as well as have better referral practices. These findings are in line with many studies nationally and internationally [26, 29–32]. A recent multicenter study involving dental pediatricians from three different countries (United States of America, Greece, and Saudi Arabia) found that knowledge was proportionally associated with experience [44]. Experience was thought to be the reason for the discrepancies seen in oral health-related knowledge and interdisciplinary practices namely referrals [45]. Experienced health professionals are expected to have more frequent exposure to cases related to oral health as well as to being more informed about loops of referrals within their institutions [9].

Participants who relied on their formal education or on the MOH as a source of oral health knowledge had greater odds of involvement in oral health-related interdisciplinary practices. The suboptimal knowledge, attitudes and practices can be mainly due to lack of organizational support both in the educational system as well as in practice. Gaps in the medical and nursing oral health-related curricula have been acknowledged in many studies [7–10, 28, 31]. It was also suggested that including oral health training in the medical staff curriculum could improve oral health knowledge and increase confidence in performing OHS and caries risk assessment [20]. The current study emphasizes the role of organizational support on interdisciplinary practices as well as on the factors that influence them.

Surprisingly, attending oral health training as part of professional development did not influence participants’ practices. Contradicting our findings some studies reported that oral health-trained physicians are more likely to provide more relevant and thorough advice to patients with oral problems, as well as more comprehensive emergency care [45–47]. Health care providers need to understand their role in the integration of oral health and the need to receive appropriate oral healthcare training. Integration of oral health into the practices of health-care providers can improve access to oral health treatment for the disadvantaged individuals. Workshops, seminars, distance learning, and in-service training with flexible timings and delivery can be some of the OHE delivery methods [40]. The Saudi Commission for Health Specialties regulates continuous medical education (CME) in Saudi Arabia and mandates all healthcare practitioners to acquire a certain number of CME hours each year to maintain their professional licensing [47]. However, this licensing body does not consider CME hours taken outside the scope of someone’s practice. We recommend the need for CME training courses to meet the learning needs for oral health especially those related to disease identification, risk assessment and prompt referrals.

In the current study participants’ interdisciplinary practices were not related to their knowledge, and attitude. The majority of the sample had an average knowledge and attitude in line with reports from Riyadh [40]. It is expected that the higher the knowledge or awareness about a condition, the more positive the attitudes about it the more likely people will be engaged into protective actions against it [48]. Participants in the current study had an average level of knowledge with essential/central/fundamental gaps in areas related to the clinical presentations of dental diseases as well as its prevention, which can explain why participants’ knowledge and attitudes did not have a greater influence on their interdisciplinary practices. Similar observations were also reported by a recent study from Eritrea as nurses’ attitudes did not affect their oral healthcare practices [49]. In addition to the macro (policies and regulations) and meso (financial compensation and manpower) level factors mentioned earlier, the daily practice of dentists and other healthcare providers are separated in KSA. In both private and public sectors dentists work in separate clinics and the various specialties are assembled in departments, for example ENT department, pediatric department etc. This may be one of the reasons that further widen the gap between dental and other healthcare providers.

There are some limitations that we would like to acknowledge. First the cross-sectional data can be interpreted only as an association rather than a cause–effect relationship. Second, the data were self-reported, so over or under-reporting may have occurred. Thirdly, there was no random sampling in the current study, which may raise the potential for selection bias in this study. Lastly, we did not look at the differences in participants’ undergraduate curricula. Despite the potential limitations mentioned, the large sample size and the validated instrument used, we believe that our findings have implications for interdisciplinary and integrated care providers worldwide as well as pave the way for further research into the effectiveness of possible solutions and interventions.

## Conclusion

The present study showed fair oral health knowledge and attitudes among participants and highlighted the presence of discrepancy between health care professionals’ IDP, knowledge, and attitudes. IDP differed among healthcare professionals. It is important to acknowledge the educational gap in oral health knowledge and provide professional training in both undergraduate and graduate courses. There is also an urgent need to build cooperative and institutional partnerships between dentists and other healthcare professionals. Lastly, simple and easily accessible accredited training and continuous education programs through distance, in-service training, workshops, and seminars should be considered by health service providers.

## Data Availability

The datasets generated and/or analysed during the current study are not publicly available due the confidentiality of the participants as some had their contact information along with comments to the research team but are available from the corresponding author on reasonable request.
